# Heme-dependent Inactivation of 5-Aminolevulinate Synthase from *Caulobacter crescentus*

**DOI:** 10.1038/s41598-018-32591-z

**Published:** 2018-09-21

**Authors:** Hiroko Ikushiro, Atsushi Nagami, Tomoko Takai, Taiki Sawai, Yuki Shimeno, Hiroshi Hori, Ikuko Miyahara, Nobuo Kamiya, Takato Yano

**Affiliations:** 1 Department of Biochemistry, Faculty of Medicine, Osaka Medical College, Osaka, 569-8686 Japan; 2 Department of Chemistry, Graduate School of Science, Osaka City University, Osaka, 558-8585 Japan; 3 Division of Diabetes and Endocrinology, Kobe University Graduate School of Medicine, Kobe, 650-0017 Japan; 4 Department of Chemistry, Graduate School of Science, Kobe University, Kobe, 657-8501 Japan; 5 The OCU Advanced Research Institute for Natural Science and Technology (OCARINA), Osaka City University, Osaka, 558-8585 Japan

## Abstract

The biosynthesis of heme is strictly regulated, probably because of the toxic effects of excess heme and its biosynthetic precursors. In many organisms, heme biosynthesis starts with the production of 5-aminolevulinic acid (ALA) from glycine and succinyl-coenzyme A, a process catalyzed by a homodimeric enzyme, pyridoxal 5′-phosphate (PLP)-dependent 5-aminolevulinate synthase (ALAS). ALAS activity is negatively regulated by heme in various ways, such as the repression of ALAS gene expression, degradation of ALAS mRNA, and inhibition of mitochondrial translocation of the mammalian precursor protein. There has been no clear evidence, however, that heme directly binds to ALAS to negatively regulate its activity. We found that recombinant ALAS from *Caulobacter crescentus* was inactivated via a heme-mediated feedback manner, in which the essential coenzyme PLP was rel eased to form the inactive heme-bound enzyme. The spectroscopic properties of the heme-bound ALAS showed that a histidine-thiolate hexa-coordinated ferric heme bound to each subunit with a one-to-one stoichiometry. His340 and Cys398 were identified as the axial ligands of heme, and mutant ALASs lacking either of these ligands became resistant to heme-mediated inhibition. ALAS expressed in *C. crescentus* was also found to bind heme, suggesting that heme-mediated feedback inhibition of ALAS is physiologically relevant in *C. crescentus*.

## Introduction

Heme, a metal porphyrin compound, is one of the most prevalent cofactors and is essential for all living organisms^1^. The first committed step in heme biosynthesis is the production of 5-aminolevulinic acid (ALA)^2^. In mammals, yeast, and the α-group of proteobacteria, ALA is formed from glycine and succinyl-CoA (the C4 pathway) via a single decarboxylating condensation reaction catalyzed by 5-aminolevulinic acid synthase (ALAS), which is a homodimeric enzyme utilizing pyridoxal 5′-phosphate (PLP) as the coenzyme. Mammals have two isozymes of ALAS: a ubiquitous housekeeping ALAS1 and an erythroid-specific ALAS2. These isozymes are synthesized as precursors in the cytoplasm and then transported into the mitochondria^3^. It is widely accepted that both isozymes of ALAS are subject to negative feedback that involves heme-dependent inhibition of a variety of processes^4^. Current evidence indicates that heme primarily regulates ALAS1 by inhibiting its mRNA expression and translation^5–7^ and by blocking the translocation of ALAS1 precursor protein into the mitochondria^8–10^. More recently, it has been reported that heme regulates the degradation of ALAS1 protein in mammalian liver mitochondria^11–14^. Despite extensive studies on heme-dependent regulation of ALAS, no convincing experimental evidence has yet been reported showing that the direct binding of heme to the presequence-processed mature form of ALAS causes the subsequent inhibition of its enzyme activity.

A number of studies on the catalytic mechanism of ALAS have been conducted^15,16^, and crystal structures of ALASs from *Rhodobacter capsulatus* and *Saccharomyces cerevisiae* have been solved^17,18^. These structures have been an excellent model for studying mammal ALASs and for predicting the relationships between gene mutations of human ALAS2 and related diseases^15–19^. To further elucidate the mechanisms of catalysis and the physiological regulation of ALAS, functional and structural analyses of ALASs from any source are valuable.

**Figure 1:**
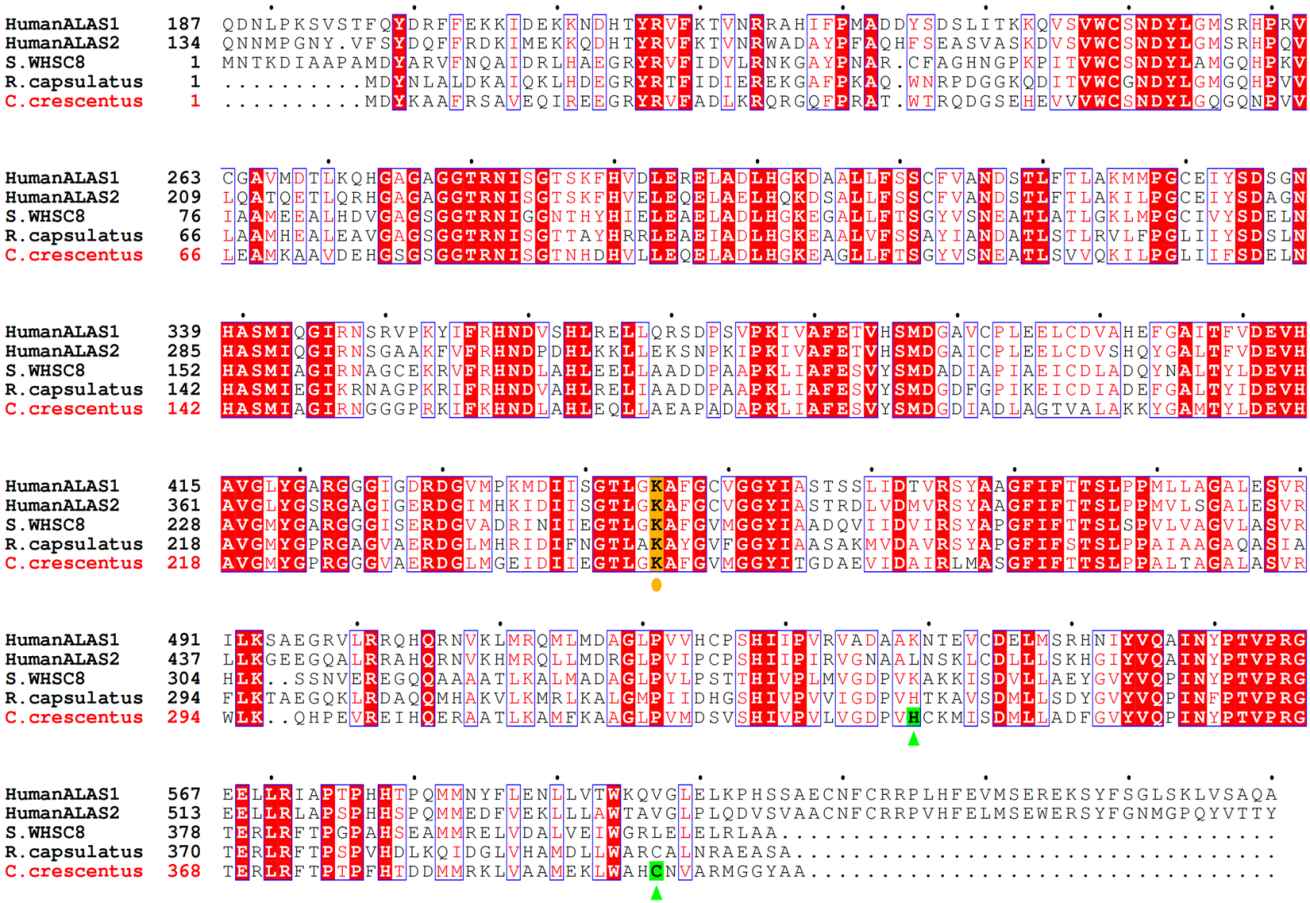
Sequence alignment of ALASs from various species. The amino acid sequence of ALAS from *C. crescentus* was compared with human ALAS1 (non-specific housekeeping isozyme), human ALAS2 (erythroid-specific isozyme), *Sphingomonas WHSC8* (*S.WHSC8*) ALAS, and *R. capsulatus* ALAS. The alignment was performed with Genetyx (Software Development Co., Fukuoka, Japan) and ESPript^58^. The lysine residues predicted to bind PLP are highlighted in yellow and marked with circles, and the residues identified as the heme axial ligands of *C. crescentus* ALAS are highlighted in green and marked with triangles. The N-terminal extending regions of 186 and 133 residues containing the presequence for human ALAS1 and ALAS2, respectively, are omitted.

In prokaryotes, ALAS-dependent heme synthesis has been found only in the α-subdivision of proteobacteria. *Caulobacter crescentus*, a member of the α-proteobacteria, is a Gram-negative, oligotrophic bacterium that grows in nutrient-poor environments such as fresh water lakes and streams^20^. The complete genome sequence of *C. crescentus* CB15 contains 4,016,942 base pairs in a single circular chromosome encoding 3,767 genes^21^. The annotated ALAS gene (CC_1355) encodes a protein of 408 amino acid residues with a predicted molecular mass of 44,273 Da. The *C. crescentus* ALAS (cALAS) is smaller than eukaryotic ALASs (typically 587–641 amino acids), and the deduced amino acid sequence of cALAS shares 48% and 49% identity with the sequences of human ALAS1 and ALAS2, respectively (Fig. 1). The *C. crescentus* genome database contains a gene (CC_3139; UniProt #Q9A3R3_CAUVC) encoding a protein with high similarity to glutamate-1-semialdehyde aminotransferase. This gene is annotated as *hemL*, the second gene of the C5 pathway of heme biosynthesis. Although it is possible that ALA may also be formed via the C5 pathway in *C. crescentus*, the *hemA* gene encoding the first enzyme of the C5 pathway, glutamyl-tRNA reductase, has not been annotated in the *C. crescentus* genome or in the genome of any other bacteria classified into the α-proteobacteria group. As Panek *et al*. have stated, it is very unlikely that the C5 pathway operates in *C. crescentus*^22^. In the present work, we set out to characterize the enzymatic nature of cALAS, and during the course of our studies, we isolated an inactive heme-bound form of cALAS.

## Results

### Overexpression and purification of *C. crescentus* ALAS

**Figure 2:**
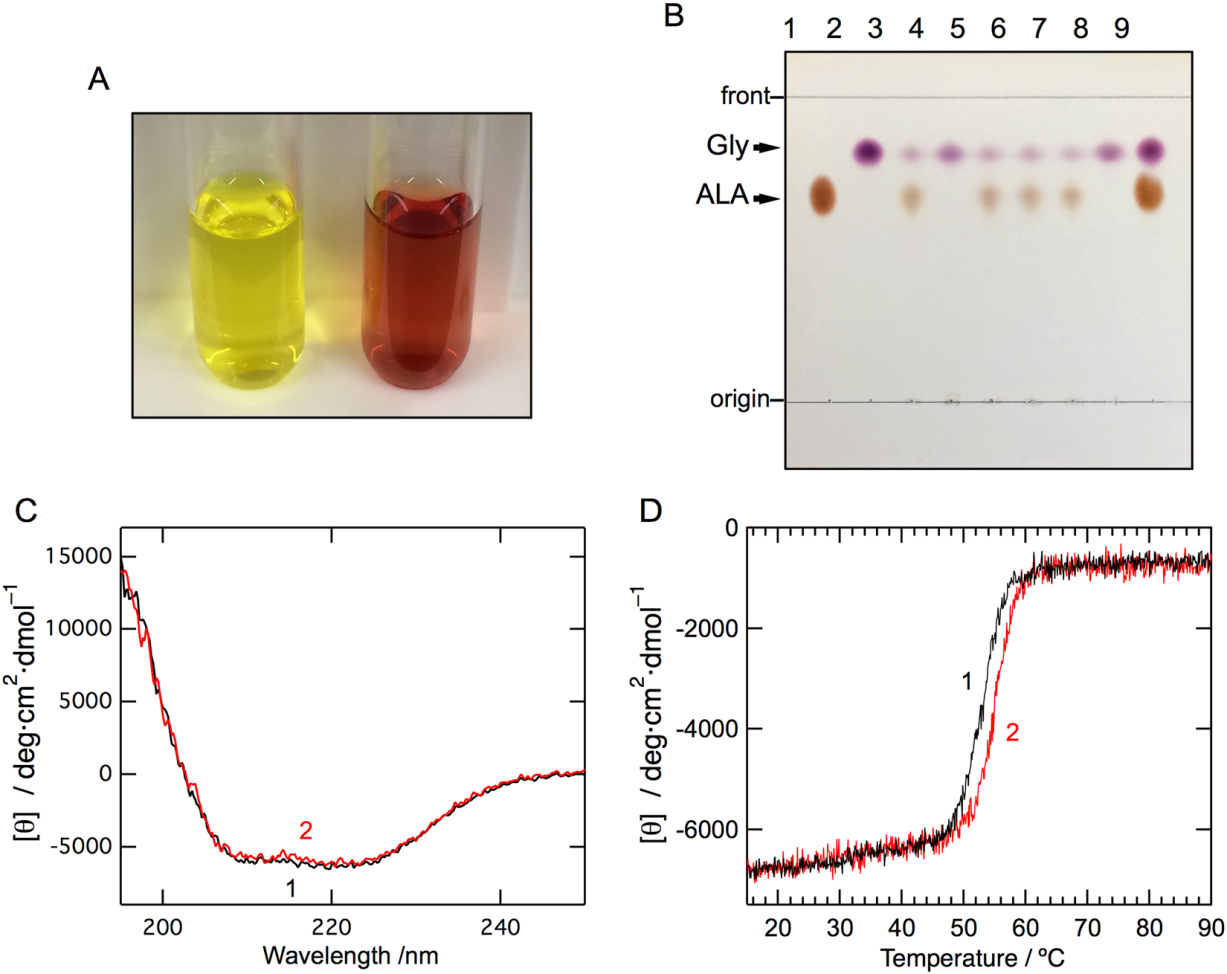
Biochemical characterization, far-UV CD spectra, and thermal stability of cALAS. (**A**) The yellow (left) and red (right) fractions after anion exchange chromatography of recombinant cALAS. (**B**) Thin-layer chromatography of the reaction products of the ALA formation assays with the purified wild-type and mutants of recombinant cALAS. ALA (lane 1) and glycine (lane 2) standards; reaction mixtures with the PLP form of the wild-type (lane 3), the heme form of the wild-type (lane 4), the PLP forms of H340A (lane 5), C398A (lane 6), and the H340A/C398A double mutant (lane 7); reaction mixture without enzyme (lane 8); and a mixture of the ALA and glycine standards (lane 9). (**C**) Far-UV CD spectra of the PLP form (line 1) and the heme form (line 2) of cALAS were measured in 50 mM sodium phosphate buffer (pH 7.4) at 20 °C. The spectra were measured at protein concentrations of 7–10 μM in a 0.2 cm light-path cell. (**D**) Thermal scan profiles of the PLP form (line 1) and the heme form (line 2). Thermal denaturation of each form of cALAS was irreversible.

The recombinant cALAS fused to a hexa-histidine-tag (His6-tag) at the C-terminus was successfully overexpressed in *Escherichia coli*. The recombinant protein was first purified with Ni-affinity chromatography. Unexpectedly, the eluate from the affinity column had a brown tinge instead of the yellow color typical for a PLP-dependent enzyme. This fraction was separated into further two fractions by anion exchange chromatography; while the first eluate was yellow (Fig. 2A, left), the second eluate was colored reddish-brown (Fig. 2A, right). The N-terminal amino acid sequence of the purified protein in each fraction was confirmed to be the same as the predicted sequence of cALAS (MDYKAAFRSAVEQ). Matrix-assisted laser desorption/ionization time-of-flight mass spectrometry produced a signal peak at m/z 44,207 for both fractions, which is in good agreement with the value of 44,273 calculated from the deduced amino acid sequence of ALAS (within experimental error). Size exclusion chromatography and native-PAGE of the yellow and red cALAS fractions indicated that both forms of cALAS were dimers in solution (*data not shown*). The red form of cALAS was also obtained when the recombinant protein without a His6-tag was overexpressed in *E. coli*, indicating that this phenomenon was not caused by the presence of the His6-tag.

### Characterization of the yellow form of cALAS

**Figure 3:**
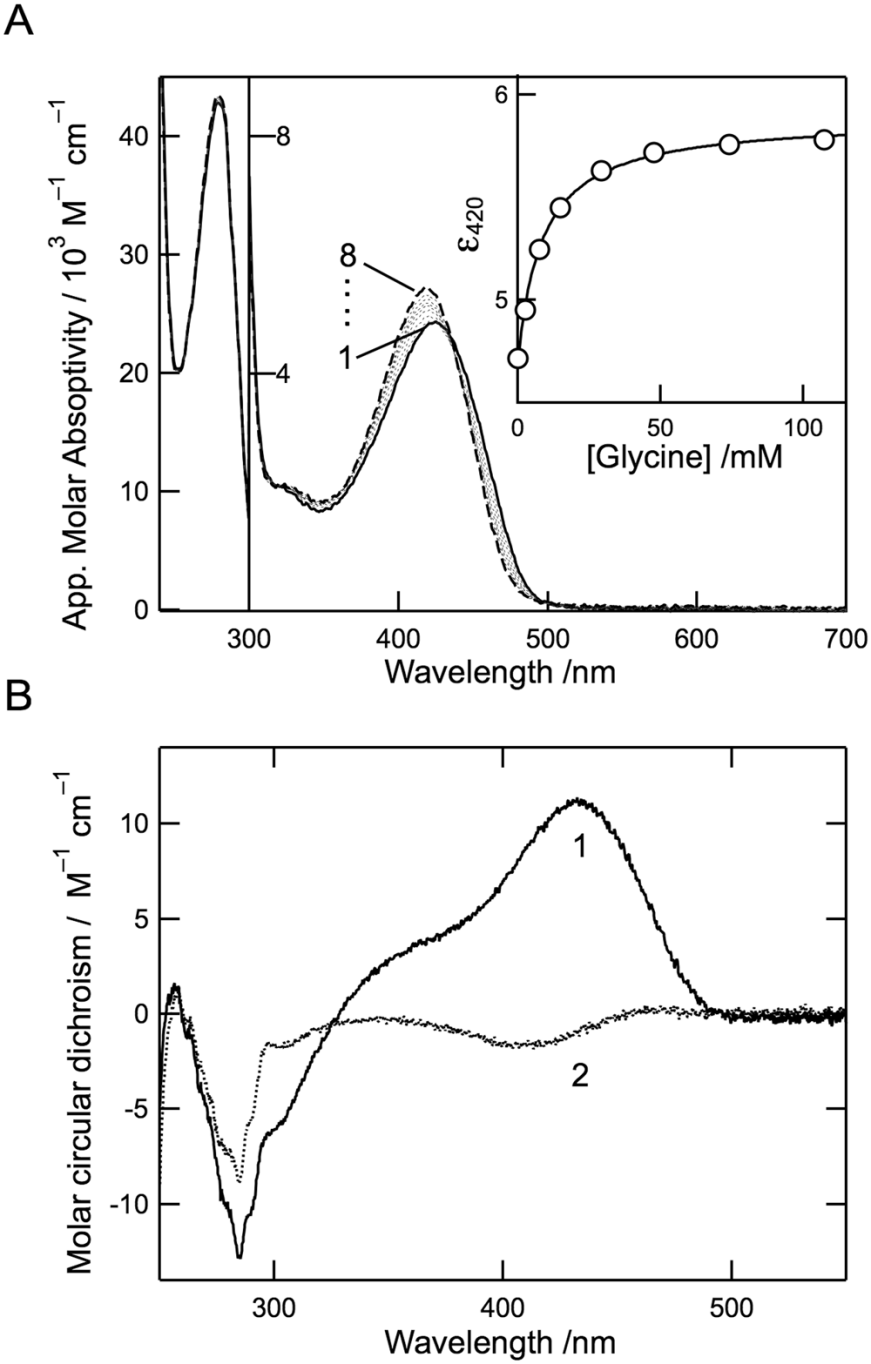
Spectroscopic characterizations of the PLP form of cALAS. (**A**) UV/Vis absorption spectra of the PLP form of cALAS (10 μM; based on monomeric state) in the presence of 0, 2.5, 7.4, 15, 29, 48, 74, and 107 mM of glycine (lines 1–8, respectively) at 25 °C. The buffer system was 50 mM HEPES-NaOH (pH 7.5) containing 150 mM KCl and 0.1 mM EDTA. The inset shows a titration curve of the molar extinction coefficient at 422 nm. (**B**) CD spectra of the PLP form of cALAS in the absence (line 1) and presence (line 2) of 100 mM glycine.

The yellow form of cALAS had a UV/Vis absorption spectrum with a single peak at 424 nm other than the protein absorption peak at 278 nm (Fig. 3A, line 1). The 424-nm peak is characteristic of PLP-dependent enzymes. In cALAS, the PLP cofactor is predicted to bind to the ε-amino group of Lys248 (Fig. 1). The absorption spectra of the yellow form of cALAS were measured in the presence of various concentrations of a substrate, glycine. Glycine binding slightly blue-shifted the 424-nm peak to 420 nm and increased its intensity (Fig. 3A, line 2–8). These spectral changes showed a hyperbolic dependency on the concentrations of glycine (Fig. 3A; inset), and the apparent dissociation constant for glycine (*K*_d_^Gly, app^) was calculated to be 10.56 ± 1.35 mM (Table 1). The circular dichroism (CD) spectrum of the yellow form of cALAS in the visible wavelength region showed a positive dichroic peak at 424 nm (Fig. 3B, line 1) corresponding to the UV/Vis absorption peak, and this CD spectrum converted to a weak negative peak at 420 nm in the presence of a saturating amount of glycine (Fig. 3B, line 2). These observations indicate that the PLP–glycine external aldimine complex was formed in the enzyme active site. All these findings are consistent with the yellow form being the canonical PLP-bound form of cALAS^23^.

**Table 1:** Kinetic parameters of ALAS enzymes for ALA formation^a^.

ALASs	${{\boldsymbol{K}}}_{{\boldsymbol{d}}}^{{\boldsymbol{Gly}},{\boldsymbol{app}}}$	${{\boldsymbol{K}}}_{{\boldsymbol{s}}}^{{\boldsymbol{Gly}}}$	${{\boldsymbol{K}}}_{{\boldsymbol{m}}}^{{\boldsymbol{Gly}}}$	${{\boldsymbol{K}}}_{{\boldsymbol{m}}}^{{\boldsymbol{SCoA}}}$	*k*_*cat*_
	mM	mM	mM	µM	s^−1^
*C. crescentus*					
Wild type	10.56 ± 1.35	12.45 ± 1.74	3.31 ± 0.45	2.42 ± 0.32	1.73 ± 0.09
H340A	11.53 ± 1.17	15.02 ± 2.73	3.52 ± 0.67	2.40 ± 0.46	1.61 ± 0.12
C398A	13.57 ± 3.10	15.63 ± 2.19	3.40 ± 0.47	2.55 ± 0.32	1.69 ± 0.07
H340A/C398A	6.46 ± 1.50	12.85 ± 2.27	3.57 ± 0.58	2.58 ± 0.39	1.79 ± 0.10
*human* ALAS2^b,c^	—	2.1 ± 0.4	15 ± 3	4.3 ± 0.6	0.030 ± 0.002
*murine* ALAS2^d^	—	8.0 ± 0.1	25 ± 4	1.3 ± 0.9	0.14 ± 0.02
*R. capsuatus* ^e^	—	—	0.25 ± 0.009	0.36 ± 0.14	0.27 ± 0.09
*R. sphaeroides* ^f,g^	—	—	0.2^e^–1.9^f^	0.5^e^–17^f^	0.12

^a^See Supplementary Information for details about data analysis and parameters. Heme-bound form has no catalytic activity as shown in Fig. 2B.

^b^Ducamp, S., *et al*.^25^.

^c^Fratz, E. J., *et al*.^26^.

^d^Lendrihas, T., *et al*.^27^.

^e^Kaufholz, A. L., *et al*.^16^.

^f^Jordan, P. M. and A. Laghai-Newton^28^.

^g^Bolt, E. L., *et al*.^29^.

The PLP form of cALAS was examined for ALA formation by incubation with two substrates, glycine and succinyl-CoA. Thin-layer chromatography (TLC) analysis of the reaction mixture yielded two ninhydrin-positive spots in the sample incubated with the purified protein (Fig. 2B, lane 3): The spots at R_f_ = 0.81 and R_f_ = 0.66 were purple and yellow, respectively, and each spot was indistinguishable in migration and color from the respective glycine and ALA standard. These results show that the PLP form of cALAS is enzymatically active in terms of ALA formation.

Steady-state kinetic analysis of the PLP form of cALAS was carried out with a continuous spectrophotometric assay utilizing α-ketoglutarate dehydrogenase (α-KGD) as described in the *Methods* section. Reciprocal plots and replots were employed to obtain *k*_cat_ and *K*_m_ values for glycine (3–40 mM) and succinyl-CoA (2–15 μM). The concentrations of succinyl-CoA were kept constant by α-KGD, which regenerates succinyl-CoA from CoA and α-ketoglutarate. As shown in Supplementary Fig. S1, the measured values were analyzed according to the ordered Bi-Bi mechanism, and the data fitted the theoretical curves well. The kinetic parameters for ALA formation were determined as follows: *K*_m_ values for glycine and succinyl-CoA and the *k*_cat_ value were 3.31 ± 0.45 mM, 2.42 ± 0.32 μM, and 1.73 ± 0.09 s^−1^, respectively (Table 1). The *k*_cat_ of cALAS was approximately 5–10 times higher than the *k*_cat_ of ALAS enzymes of other organisms. Unlike murine ALAS2^24^, the lines in the reciprocal plots intersected above the horizontal axis, showing that the cALAS dissociation constant for glycine (*K*_s_^Gly^ = 12.45 mM) was greater than the *K*_m_ for glycine. This value for *K*_s_^Gly^ showed good agreement with the value of the apparent dissociation constant for glycine (*K*_d_^Gly,app^ = 10.56 mM) determined with the spectrophotometric titration experiment (Fig. 3A). The *K*_m_ for glycine was higher than that for succinyl-CoA by three orders of magnitude. A similar tendency has been observed in ALASs from other organisms such as the human, mouse, and *Rhodobacter sphaeroides*^16,25–29^.

### Characterization of the red form of cALAS

**Figure 4:**
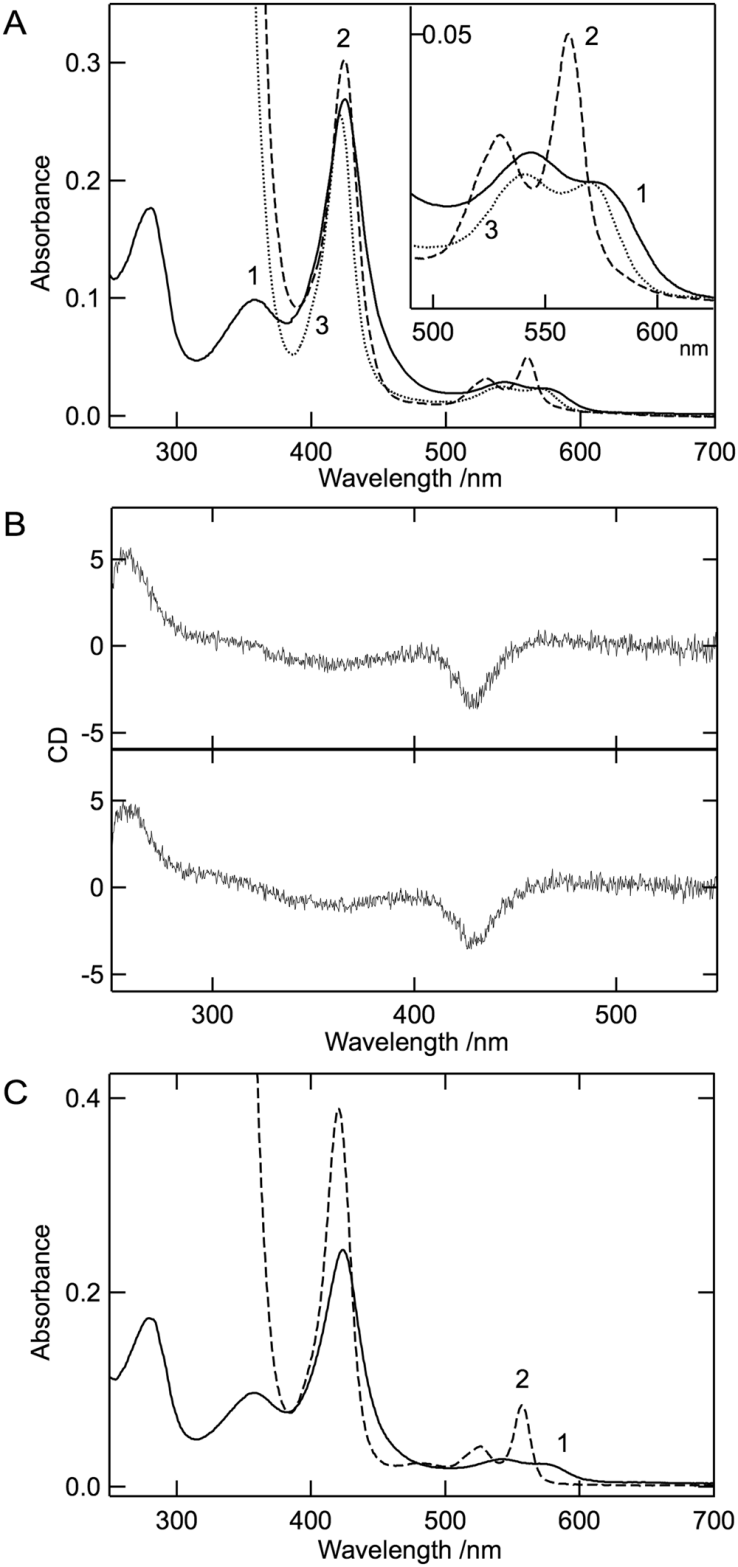
Spectroscopic characterization of the heme form of cALAS. (**A**) UV/Vis absorption spectra of the heme form of purified cALAS (solid line (line 1)), the reduced form (dashed line (line 2)), and the reduced CO-bound form (dotted line (line 3)). The inset shows an enlarged image in the region 490–625 nm. (**B**) CD spectra in the visible region for the heme form of cALAS. CD spectra of the heme form of cALAS were measured in the absence (upper) and presence (lower) of 100 mM glycine. (**C**) The absorption spectra of the heme-form of cALAS (line 1) and the dithionite-reduced pyridine-hemochrome (line 2). For A–C, the buffer system was 50 mM HEPES-NaOH (pH 7.5) containing 150 mM KCl and 0.1 mM EDTA. The measurements were performed at 25 °C at a protein concentration of 3 μM (based on monomeric state). The concentration of heme was estimated from line 2 of panel C was 2.6 μM.

The red fraction of the purified cALAS was free from contaminating proteins as judged by SDS-PAGE and mass spectrometry. There were no detectable differences in the far-UV CD spectra between the red form and the yellow form, indicating that their gross conformations were almost identical (Fig. 2C). The thermal stability of each form of cALAS was assessed by temperature-scanning the CD at 220 nm (Fig. 2D). The heat-induced denaturation process was irreversible in both cases. The melting temperatures were almost the same; Tm = 53 °C for the PLP form and Tm = 54 °C for the red form. The absorption spectrum of the red form was typical of a heme-containing protein such as cytochrome P450 or cytochrome b_5_ (Fig. 4A)^30^. The CD spectrum of the red form in the visible wavelength region showed a negative Cotton band at 423 nm corresponding to the Soret peak of the UV/Vis absorption spectrum, and this band was unchanged by the addition of excess glycine (Fig. 4B). ALA formation was not detected for the red form of cALAS with TLC analysis (Fig. 2B; lane 4). In contrast to the PLP form of cALAS, the red form of cALAS was enzymatically inactive.

Further spectroscopic analysis of the red form of cALAS was performed to characterize the nature of the bound heme and its binding mode. The UV/Vis absorption spectrum of the red form of cALAS showed an intense Soret peak (γ-peak) at 424 nm coupled with a δ-peak at around 360 nm, an α-band shoulder at around 568 nm, and a more intense β-peak at 540 nm (Fig. 4A, line 1 (solid line)). This is typical of the ferric state (Fe^3+^ oxidation state) spectrum and closely resembles that of *b*-type cytochrome^31,32^, in which two amino acid residues contribute to binding the heme iron. In contrast, the heme of P450 is coordinated by cysteine thiolate and a weakly bound water molecule to show a Soret peak at around 415 nm^33,34^. The nature of the red form of cALAS is consistent with a low-spin six-coordinate ferric heme with axial ligands comprising a thiolate ligand trans to a neutral donor such as histidine, as observed in cystathionine β-synthase (CBS)^35^, HRI^36^, Bach1^37^ and RcoM-2^38^. When the sample was treated with sodium dithionite, the spectrum was converted into that of the reduced heme chromophore with distinct α-, β-, and γ-peaks at 560, 530, and 424 nm, respectively (Fig. 4A, line 2 (dashed line)). The addition of carbon monoxide (CO) to the reduced form changed the spectra into a CO-bound form with α-, β-, and γ-peaks at 570, 540, and 420 nm, respectively (Fig. 4A, line 3 (dotted line)). Unlike P450 or CBS which shows CO-liganded reduced spectra with an intense peak at 450 nm reflecting cysteine thiolate–Fe (ferrous)–CO, the Soret peak at 420 nm is consistent with the loss of the thiolate ligand, and the resulting species is presumed to be hexa-coordinated with the axial ligand of histidine nitrogen–Fe (ferrous)–CO. Hereafter, the inactive red form of cALAS is referred to as the heme form of cALAS in contrast to the active PLP form.

A reduced pyridine hemochrome assay was used to determine whether the chromophore associated with cALAS was *b*-type heme. The spectrum of the heme form of cALAS after the treatment was identical to that of the pyridine hemochrome derived from hemin, displaying peaks at 418, 524, and 557 nm (Fig. 4C line 2 (dashed line)). The heme content of cALAS was calculated from the absorbance at 557 nm and ranged between 17.3–23.5 nmol heme/mg cALAS. Because the theoretical value for the cALAS subunit is 22.6 nmol/mg protein, the heme content is in good agreement with a one-to-one stoichiometric ratio of heme to subunit of the cALAS dimer.

Additional evidence for the thiolate ligation of ferric heme in cALAS came from analysis of the electron paramagnetic resonance (EPR) spectra (Supplementary Fig. S2). The EPR spectrum of cALAS at 15 K showed typical low-spin P-type signals with g-values of 2.46 (g_1_), 2.26 (g_2_), and 1.88 (g_3_). The g-value of 4.44 was interpreted as the non-heme iron signal derived from a degradation product of heme. The values for g_1_–g_3_ suggested that the axial ligands of cALAS heme iron are a histidine-donated nitrogen and a cysteine/methionine-donated sulfur (N–Fe–S) rather than bis-histidine ligands^39,40^. The high-spin EPR signal of cALAS with a g-value of 6.03 was barely detected at 5 K (*data not shown*), and this result further supported the above conclusion. The fact that the heme form lacking a His6-tag showed essentially the same UV/Vis and EPR spectra as the tagged cALAS excludes the possibility that the heme is nonspecifically bound to an imidazole group contained in the His6-tag of the recombinant protein.

### Identification of the heme-binding site in cALAS

The spectroscopic properties of the heme form of cALAS strongly suggested that the axial ligands of the bound heme were cysteine/methionine and histidine residues. To identify the ligand residues, alanine scanning mutagenesis was performed on 35 candidate residues (3 cysteines, 15 histidines, and 17 methionines) of cALAS (Fig. 1). All the alanine-substituted mutants were successfully expressed in *E. coli* and purified (*data not shown*). Only two mutants, H340A and C398A, were yellow, instead of the reddish color, typical of the PLP-bound enzyme. The double mutant H340A/C398A was then constructed and yielded the yellow, PLP form of cALAS. All three mutants retained enzyme activity, and their kinetic parameters were essentially the same as those of the wild type enzyme (Fig. 2B, lanes 5–7, and Table 1). These results clearly show that His340 and Cys398 are the axial ligands of heme in cALAS.

### Binding mode and stoichiometry of chromophores of cALAS

**Figure 5:**
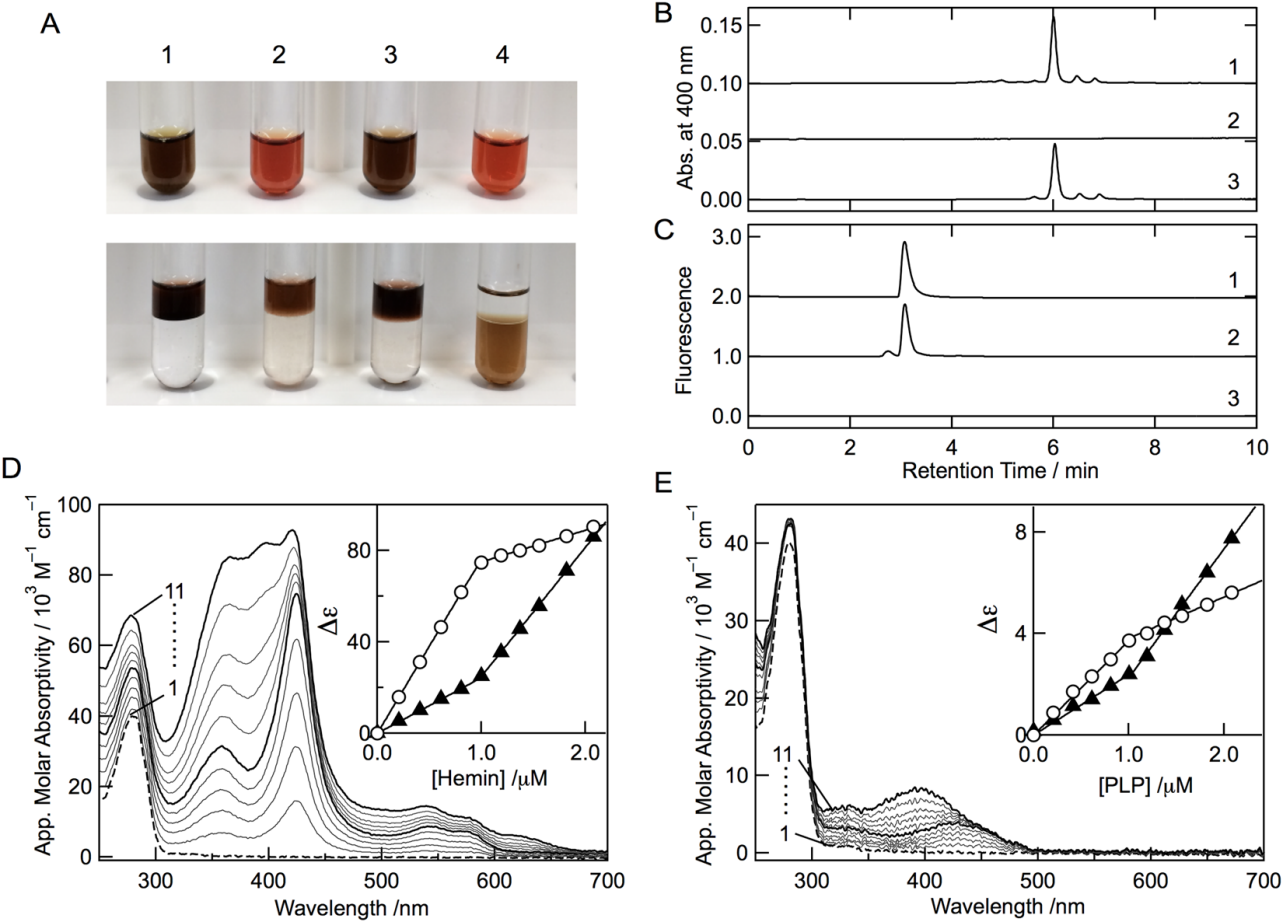
Analysis of chromophores from purified cALAS. (**A**) Acidic-butanone extraction of the chromophores of hemin (1), the heme form of cALAS (2), equine heart myoglobin (3), and equine heart cytochrome c (4). The solutions are shown before (upper) and after (lower) extraction, respectively. Non-covalently bound heme was extracted into the upper organic layer. (**B**) HPLC analysis of heme in the acidic-butanone-extracts of hemin (line 1), PLP-bound cALAS (line 2), and heme-bound cALAS (line 3). (**C**) HPLC analysis of PLP in deproteinized extracts of the PLP control (line 1), PLP-bound cALAS (line 2), and heme-bound cALAS (line 3). (**D**) Hemin titration of the apoenzyme. Spectral changes of D214A apo-cALAS (1 μM; based on monomeric state) upon addition of hemin. Lines 1–11 correspond to 0, 0.21, 0.41, 0.61, 0.81, 1.00, 1.19, 1.37, 1.56, 1.82, and 2.08 μM hemin, respectively. The inset shows changes in the apparent molecular absorptivity at 425 nm (open circles) and 385 nm (closed triangles) at these concentrations of hemin. (**E**) PLP titration of the apoenzyme. Spectral changes of D214A apo-cALAS (1 μM; based on monomeric state) upon addition of PLP. Lines 1–11 correspond to 0, 0.21, 0.41, 0.61, 0.81, 1.00, 1.19, 1.37, 1.56, 1.82, and 2.08 μM PLP, respectively. The inset shows changes in the apparent molecular absorptivity at 424 nm (open circles) and 385 nm (closed triangles) at these concentrations of PLP.

To establish whether the chromophore of the heme form of cALAS covalently binds to the protein, acidic butanone extraction was carried out. In the control experiments using hemin, myoglobin, and cytochrome c, the protein-free hemin and the non-covalently bound heme of myoglobin were completely extracted to the organic layer, whereas the covalently bound heme of cytochrome c was not extracted at all and remained in the aqueous layer (Fig. 5A). Acidic butanone treatment of the heme form of cALAS released the chromophore from the protein; the reddish brown organic layer separated from the colorless lower layer of the aqueous phase that contained the apo-protein. The extracted chromophore from the heme form of cALAS was analyzed by high-performance liquid chromatography (HPLC). A peak at the retention time of 6 min corresponding to heme B was detected, but peaks of other heme derivatives such as heme O and heme A were not detected (Fig. 5B). Furthermore, whereas a peak for PLP was observed for the PLP form of cALAS, PLP was not detected for the heme-bound form of cALAS (Fig. 5C).

Both chromophores bound to the protein so tightly that they could not be removed from cALAS under non-denaturing conditions. The purified PLP form of cALAS could also not be converted to the heme form by the addition of hemin *in vitro*. Hemin added in large excess turned the color of the sample to brown, but the absorption spectrum of the sample was that of free heme overlaid with the PLP form. This indicates that the added heme did not bind specifically to the purified PLP form of cALAS. Similarly, the purified heme form of cALAS could not be converted to the PLP form by the addition of an excess amount of PLP.

To determine the stoichiometry of chromophore binding to the cALAS subunits, Asp214 of cALAS, which was assumed to be a PLP-anchoring site, was replaced with alanine. The apo-protein was prepared from the PLP form of D214A cALAS by cysteine treatment and dialysis, and titration experiments with both hemin and PLP were then performed (Fig. 5D,E). Upon titration with hemin, D214A cALAS displayed a growing Soret band at 425 nm and a concomitant increase in absorption at around 540 and 570 nm (β and α bands of heme). These spectral features are identical to the characteristics of the heme form of cALAS and are easily distinguished from the absorption of free heme. Addition of heme beyond saturation caused an increase in absorbance at around 385 nm, representing the accumulation of non-coordinated heme. To determine the concentration at which cALAS becomes saturated with heme, the absorbance of cALAS-heme at 425 and 385 nm was plotted against the hemin concentration. The inflection point on this plot indicated that the protein (1.0 µM apo-cALAS based on monomeric state) was saturated at 1.0 µM heme (Fig. 5D, inset), consistent with a 1∶1 heme to cALAS stoichiometry, suggesting that each cALAS subunit contains a single heme-binding site. The cALAS reconstituted with heme showed a bright red color.

In the same manner, upon PLP titration, cALAS displayed spectral changes supporting internal Schiff-base formation between PLP and Lys248. Addition of PLP beyond saturation shifted the absorption maximum toward 385 nm, representing the accumulation of unbound PLP. To obtain the concentration at which cALAS becomes saturated with PLP, the absorbance of the PLP-titrated sample at 425 and 385 nm was plotted against the PLP concentration. The inflection point indicated that saturation of the protein (1.0 µM apo-cALAS based on monomeric state) was reached at 1.0 µM PLP (Fig. 5E, inset), consistent with a 1∶1 PLP to cALAS stoichiometry. The cALAS reconstituted with PLP showed a yellow color.

### Interconversion between the PLP form and the heme form of cALAS in the *E. coli* lysate

Although interconversion between the PLP form and heme form of cALAS was not detected in the purified enzyme system, addition of 0.2 mM of PLP to the cALAS-overexpression *E. coli* lysate before the first chromatography step increased the yield of the PLP form several fold. We also examined the effect of hemin supplementation on the formation of the heme form in the cALAS-overexpression *E. coli* lysate. When 0.05–0.1 mM hemin was added to the *E. coli* lysate after cell disruption, almost all the overproduced cALAS was recovered in the heme form. These results suggest that the interconversion between the PLP form and heme form of cALAS may be dependent on specific endogenous protein factors in *E. coli*, although *E. coli* is a member of the γ-proteobacteria.

### Expression of recombinant cALAS in *C. crescentus* CB15 cells

**Figure 6:**
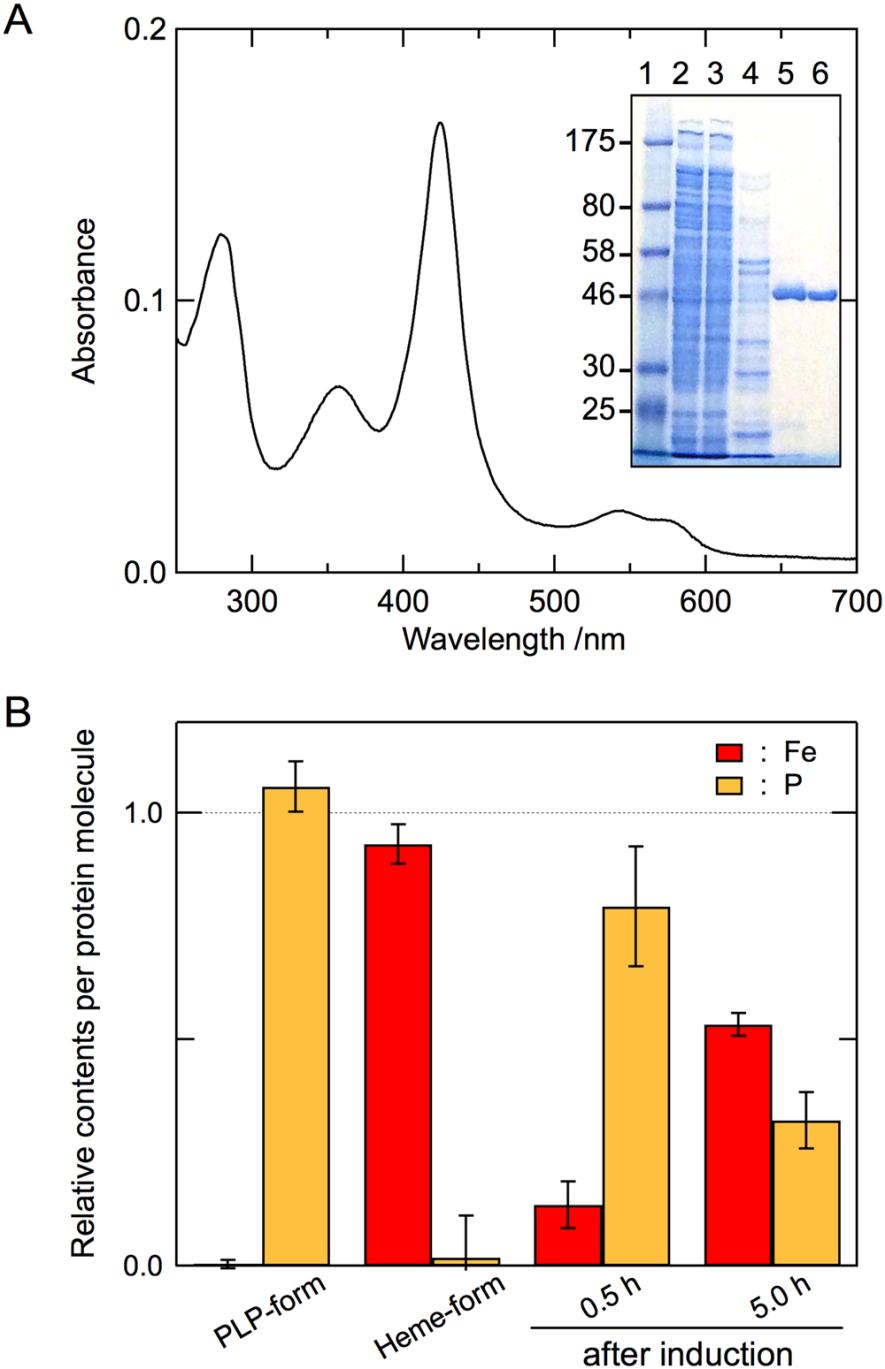
cALAS expression in *C. crescentus*. (**A**) The absorption spectrum of the heme form of cALAS expressed in *C. crescentus* was measured under the same conditions described in Fig. 4. The *inset* shows SDS-PAGE at various steps of the purification of cALAS from *C. crescentus* cells. Lane 1, molecular weight marker (NEB #P7703); lane 2, crude extract of *C. crescentus* cells expressing His6-tagged cALAS; lane 3, flow-through fraction from the Ni-affinity column; lane 4, wash-out fraction with 60 mM imidazole from the affinity column; lane 5, elution fraction with 200 mM imidazole from the affinity column; lane 6, cALAS eluted from the Mono Q anion exchange column. Samples containing ~1 μg of protein/lane were further analyzed. (**B**) The contents of iron (derived from heme) and phosphorus (derived from PLP) relative to sulfur (derived from sulfur-containing amino acid residues of cALAS) were analyzed by ICP-AES. Because cALAS contains 20 sulfur-containing amino acid residues (3 cysteines and 17 methionines), 20 μM heme, 20 μM PLP, and 400 μM Cys/Met mixture (3:17 molar ratio) were utilized as the calibration standards for iron, phosphorus, and sulfur. Enzyme preparations were performed in triplicate, and three measurements were carried out for each sample. The experimental errors for each sample were within ±5%. Yellow and red bars show phosphorus and iron, respectively. From left to right, the purified PLP form and heme form expressed in *E. coli*, and the affinity-purified samples expressed in *C. crescentus* 30 min and 5 h after induction.

To evaluate whether the heme form of cALAS is an artifact of heterologous expression in *E. coli*, we examined this chromophore in cALAS expressed in *C. crescentus*^41^. The induction of protein expression caused neither a noticeable phenotypic change nor growth inhibition of the transformed bacteria during cultivation. The expression level of cALAS in *C. crescentus* was much lower than that in *E. coli*, but the His6-tagged recombinant protein was still homogeneously purified from *C. crescentus* as judged by SDS-PAGE (Fig. 6A, inset). The purified sample exhibited the characteristic features of a heme protein: a sharp Soret peak at 424 nm with a shoulder at 363 nm and a broad band around 550 nm (Fig. 6A). These absorption maxima of cALAS purified from *C. crescentus* were the same as those of the cALAS expressed in *E. coli*.

The amounts of iron and phosphorus atoms per protein molecule were examined by inductively coupled plasma atomic emission spectroscopy (ICP-AES) to analyze the change in the relative amounts of the PLP and heme forms of cALAS after induction of His6-tagged cALAS expression (Fig. 6B). Ni-affinity-purified cALAS expressed in *C. crescentus* was used, and the protein expression experiments were performed in triplicate. In the initial phase of cALAS expression (0.5 h after induction), the relative contents of phosphorus and iron were 79.4 ± 13.3% and 13.4 ± 5.2%, respectively, indicating that the majority of the expressed cALAS was in the PLP form; 4.5 h later, the phosphorus and iron contents were 32.1 ± 6.2% and 53.3 ± 2.5%, respectively, showing that the proportion of the heme form had exceeded that of the PLP form.

These results indicate that a heme-bound form of cALAS occurs in *C. crescentus* and that the PLP form converts to the heme form as heme accumulates in the cells.

## Discussion

Unexpectedly, cALAS was purified as two forms of cofactor-bound enzyme: the PLP-bound active form and the heme-bound inactive form. The only example of an enzyme that binds PLP and heme simultaneously as cofactors is CBS, which catalyzes the condensation of L-serine and L-homocysteine to give L-cystathionine. Human CBS contains low-spin six-coordinate ferric heme with axial ligands comprising the sulfhydryl group of Cys52 and the Nε2 atom of His65^42^. The coexistence of heme and PLP on the protein is necessary to maximize enzyme activity, and the loss of heme decreases the enzyme activity to 25%. It has been proven, however, that the heme does not participate directly in the PLP-mediated enzymatic mechanism of CBS. Although the crystal structure of the heme-bound form of CBS has been determined, the exact function of the heme in CBS remains unknown. It has been considered that the heme plays a regulatory role in redox sensing and/or enzyme folding in concert with *S-*adenosyl-L-methionine, an allosteric activator of CBS^43,44^. In contrast to CBS, cALAS could not bind these two cofactors simultaneously, and the heme form of cALAS neither contained the essential cofactor PLP nor exhibited enzyme activity. All cALAS mutants, in which either one or both of the axis-ligand residues for heme were replaced with alanine, completely lost heme-binding capacity and were as active as the wild-type, PLP form of cALAS. Heme-dependent inactivation of cALAS would result in the suppression of an oversupply of heme in the cells. Thus, an obvious interpretation of the present results is that cALAS activity is subjected to feedback inhibition by the direct binding of heme to the enzyme.

**Figure 7:**
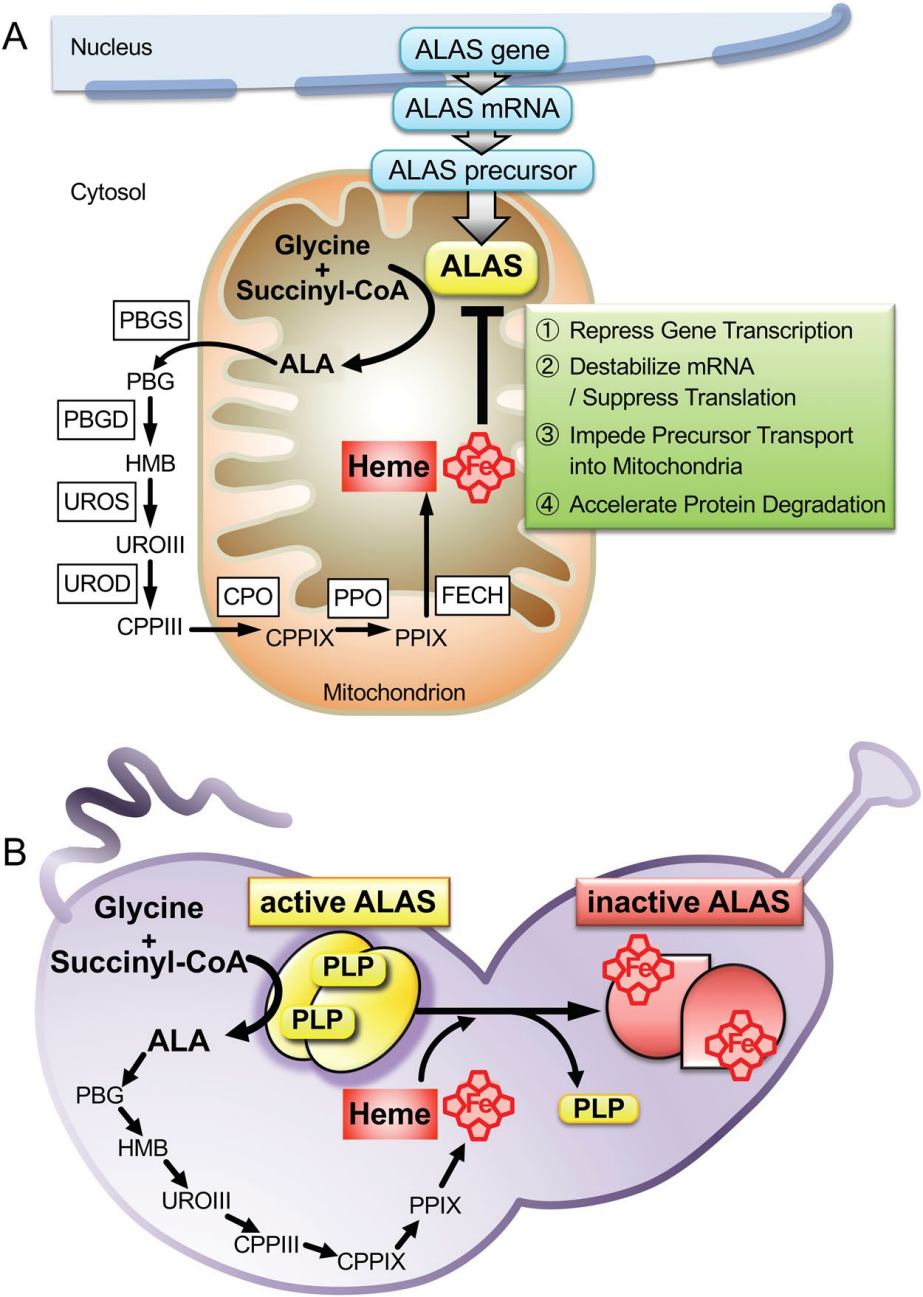
Heme-dependent feedback inhibition of ALAS in the heme biosynthetic pathway. (**A**) In the mammal cells, heme primarily regulates ALAS1 by inhibiting its mRNA expression and translation and by blocking the translocation of ALAS1 precursor protein into the mitochondria. Heme promotes the degradation of ALAS1 protein in mitochondria. (**B**) The heme biosynthesis in *C. crescentus* is negatively regulated via inactivation of cALAS by direct heme binding, where the excess free heme directly binds to each subunit of cALAS homodimer in one-to-one stoichiometry to releases an essential coenzyme PLP and is inactivated quickly, thus providing a mechanism for bacteria to react to the toxic unbound tetrapyrrole products. Abbreviations: PBG; porphobilinogen, HMB; hydroxymethylbilane, URO III; uroporphyrinogen III, CPP III; coproporphyrinogen III, CPPIX; coproporphyrinogen IX, PPIX; protoporphyrinogen IX, PBGS; porphobilinogen synthase, PBGD; porphobilinogen deaminase, UROS; uroporphyrinogen III synthase, UROD; uroporphyrinogen III decarboxylase, CPO; coproporphyrinogen III oxidase, PPO; protoporphyrinogen IX oxidase, FECH; ferrochelatase.

It is known that ALAS activity in mammals is negatively regulated by heme in various ways (Fig. 7A). It has been reported that heme affects ALAS1 by repressing gene transcription^5^ or mRNA translation^7^, by accelerating mRNA degradation^6^, and by impeding mitochondrial translocation of the precursor protein^10^. The three cysteine-proline-rich (CP) motifs in the leader sequence of ALAS1 are similar to the heme regulatory motifs (HRMs) of the yeast transcriptional activator HAP1, which binds heme via the HRMs. The CP motifs in ALAS1 precursors are responsible for heme-mediated inhibition of the mitochondrial translocation of the precursor^10^. Although it is not known whether heme binds directly to the mature form of ALAS to control enzyme activity, it has been reported that heme accelerates the degradation of mature ALAS1 protein in mammalian liver mitochondria^11–14^. However, significant degradation of cALAS was not detected during the expression, purification, or storage of the heme form. Indeed, the overall structure of the heme form of cALAS was very similar to that of the PLP form in terms of the oligomerization state, the gross protein structure (far-UV CD spectra), and thermostability.

After induction in the native host *C. crescentus*, the heme-cALAS to PLP-cALAS ratio was observed to increase in a time-dependent manner, indicating that these findings are physiologically relevant. Because the overproduction of free heme in cells would exert toxic side effects, either the excess heme needs to be immediately removed, or ALA formation should be suppressed. It would be important for *C. crescentus* to economize energy consumption in a nutrient-poor habitat by reducing the protein degradation/resynthesis cycle. The inactivation of cALAS by direct heme binding may be beneficial for the regulation of heme biosynthesis in *C. crescentus*; when heme levels become excessive, the free heme binds to cALAS, and this heme-bound form of cALAS is transiently inactivated by the release of PLP without the need for protein degradation (Fig. 7B). When there is a shortage of heme, the heme is released from cALAS to restore the active PLP-bound holo-enzyme.

The heme-binding site reported here is located far from the PLP-binding active site in the structure of *R. capsulatus* ALAS^17^. The C-terminal region (Arg399–Ala410) of *R. capsulatus* ALAS, containing the Cys400 residue that corresponds to Cys398 of cALAS, was disordered in the crystal structure of the PLP-bound holo-enzyme, indicating that this region is highly flexible. Upon heme binding, the flexible C-terminal region would therefore become structurally fixed to the α-helix containing His340 by forming the Cys398–Fe–His340 linkage. This heme-mediated fixation should induce rearrangements in the protein structure that are propagated to the active site, resulting in the loss of PLP-binding capacity. Heme-dependent control of protein function has been reported in several proteins^45,46^, such as the transcription repressor Bach2^47,48^, the regulatory sulphonylurea receptor subunits SUR2A of the ATP-sensitive potassium ion channel K_ATP_^49^ and the A-type potassium ion channel Kv1.4^50^, in which the heme-sensing/binding site is a cysteine residue other than the CP motif. And it has also been pointed out that their heme-sensing/binding sites being originally unstructured alter their conformation to a compact form upon the heme-binding.

The α-proteobacteria are divided into twelve orders (*Caulobacterales*, *Kiloniellales*, *Kopriimonadales*, *Kordiimonadales*, *Magnetococcales*, *Parvularculales*, *Rhizobiales*, *Rhodobacterales*, *Rhodospirillales*, *Rickettsiales*, *Sneathiellales*, and *Sphingomonadales*). The heme-binding His/Cys motif reported here is completely conserved in ALASs from all species of *Caulobacterales*. However, the majority of α-proteobacteria ALASs do not contain this heme-binding motif. ALASs from some species of *Rhodobacterales*/*Rhizobiales* appear to contain the corresponding His/Cys residues—for example, His342 and Cys400 of *R. capsulatus* ALAS (Fig. 1). To determine whether *R. capsulatus* ALAS also binds heme, recombinant *R. capsulatus* ALAS was expressed in *E. coli* and purified. Both the heme- and PLP-bound forms were obtained, as for cALAS (*data not shown*). This further supports our hypothesis that the His/Cys residues are indeed a bona fide heme-binding motif conserved in some species of α-proteobacteria. We are now attempting X-ray crystallographic studies of both the PLP and heme forms of recombinant cALAS. The structure of the heme form would clarify the heme binding mode and the mechanism of heme-mediated inactivation of enzyme activity. Although interconversion of the PLP and heme forms of cALAS could not be reproduced by the addition of these cofactors to the purified enzyme, this was achieved in the *E. coli* lysate. It is likely that the process of binding/dissociation of heme to/from cALAS is not a simple passive event but requires specific (energy-dependent) protein machinery contained in the lysate, and such machinery may also operate in *C. crescentus*.

## Materials and Methods

### Source and materials

Succinyl-CoA was obtained from Funakoshi (Tokyo, Japan). Hemin and cumate (4-isopropylbenzoic acid) were from Sigma-Aldrich. These and all other chemicals were of the highest grade commercially available.

### Overexpression and purification of cALAS recombinant proteins

The gene encoding cALAS (CC_1355; UniProt #Q9A8J8_CAUCR) was PCR-amplified from *C. crescentus* genomic DNA (ATCC 19089D). *E. coli* BL21(DE3) pLysS cells were transformed with the expression plasmid pET-28b (Novagen, Madison, WI) carrying the cALAS gene, and protein expression was induced at 37 °C for 4 h with isopropyl 1-thio-β-D-galactopyranoside at a final concentration of 0.2 mM. Expression of cALAS in *C. crescentus* (*C. vibrioides* Henrici and Johnson; ATCC 19089) was achieved with the cumate-inducible expression system^41^ using pQF plasmids (Addgene, Cambridge, MA) carrying the cALAS gene.

The cell lysates were prepared by sonication and centrifuged (15,000 × g) for 30 min at 4 °C. The His6-tagged cALASs were purified with a two-step procedure comprising column chromatography with a HisTrap FF Crude column (GE Healthcare, Pittsburgh, PA) followed by a Mono Q 5/50GL column using the ÄKTA Fast Protein Liquid Chromatography system (GE Healthcare), all according to the manufacturer’s instructions. Protein concentrations were calculated based on absorption at 280 nm using the molar extinction coefficient of 42,740 M^−1^ cm^−1^ for the purified PLP-bound enzyme. Approximately 100 mg of purified cALAS protein were obtained from 2 L of *E. coli* culture, and ~2 mg of purified cALAS were obtained from 2 L of *C. crescentus* culture.

Site-directed mutagenesis was performed with the QuikChange Lightning Site-Directed Mutagenesis kit (Agilent, Santa Clara, CA) according to the prescribed protocol. Each mutant cALAS was expressed and purified in the same way as the wild-type enzyme.

The apo-form of D214A cALAS was prepared by incubation with 200 mM L-cysteine in 200 mM sodium phosphate buffer (pH 7.4) at 37 °C for 6 h. The enzyme was then dialyzed twice against 1 L of 200 mM sodium phosphate buffer (pH 7.4) at 4 °C.

### Spectral measurements

UV/Vis spectra of cALAS were recorded with a Hitachi U-3310 spectrophotometer at 25 °C. The buffer solution for the spectrometric measurements contained 50 mM HEPES-NaOH (pH 7.4), 150 mM KCl, and 0.1 mM EDTA. Circular dichroism (CD) spectra and thermal scans were recorded on a Jasco J-720-WI spectropolarimeter. Far-UV (190–250 nm) and near-UV (250–310 nm) CD measurements were performed at 20 °C. For thermal scans, the protein samples (0.2 mg/mL) were heated from 10 to 95 °C and subsequently cooled to 10 °C with a heating/cooling rate of 1 °C/min controlled by a Jasco programmable Peltier element.

Electron paramagnetic resonance (EPR) measurements were carried out at X-band (9.23 GHz) microwave frequency using a Varian E-12 EPR spectrometer with 100-kHz field modulation. The microwave frequency was calibrated with a microwave frequency counter (model TR5212; Takeda Riken Co., Ltd.). The strength of the magnetic field was determined with an NMR field meter (model EFM 2000AX; Echo Electronics Co., Ltd.). An Oxford flow cryostat (ESR-900) was used to achieve temperatures down to 15 K and 5 K.

### Thin-layer chromatography (TLC) analysis of the cALAS-dependent reaction product

Each purified cALAS (4.5 nmol) was incubated with 1.0 mM glycine and 0.5 mM succinyl-CoA in 100 μL of 50 mM HEPES-NaOH pH 7.4 containing 150 mM KCl at 37 °C for 1 h. A 5 μL aliquot of each reaction mixture was applied to a RP-18 WF_254_s HPTLC plate (Merck) and developed in a methanol-water-TFA (60:40:0.001 v/v) solvent system. The separated spots on the TLC plate were visualized with ninhydrin spray and heating at 100 °C for 1 min.

### Steady-state enzyme assay

ALAS activity was determined at 25 °C using a continuous spectrophotometric assay coupled with α-ketoglutarate dehydrogenase (α-KGD)^51^. In this method, the α-KGD couples the regeneration of CoASH to succinyl-CoA to the reduction of NAD^+^ to NADH. The production of NADH is equivalent to the production of ALA and could be followed spectrophotometrically at 340 nm. Reaction mixtures contained 50 mM HEPES-NaOH pH 7.4, 0–15 μM succinyl-CoA, 0–40 mM glycine, 1 mM α-ketoglutarate, 1 mM NAD^+^, 3 mM MgCl_2_, 0.25 mM thiamine pyrophosphate, 0.42 units α-KGD, and 20 μg recombinant cALAS in a total volume of 1.0 mL. Data analysis according to the kinetic model of the ordered Bi-Bi mechanism is provided in Supplemental Information.

### Other Methods

Heme concentration was determined from the absorbance of the α-peak of the reduced pyridine hemochrome using *ε*_557_ = 32.0 mM^−1^ cm^−1^ ^52,53^. Heme extraction with acid-butanone was carried out as described by Mrázová^54^. PLP and heme bound to the purified cALAS were analyzed using reverse-phase HPLC^55,56^. The iron, phosphorus, and sulfur contents in the enzyme preparations were determined by ICP-AES (Thermo iCAP 6300). The protein concentrations during the purification steps were determined with a Bio-Rad Protein Assay Kit (Bio-Rad Laboratories, Hercules, CA) using bovine serum albumin as the standard. SDS-polyacrylamide gel electrophoresis (SDS-PAGE) was carried out with an SDS-Tris system using 10% polyacrylamide gel according to the procedure described by Laemmli^57^.

## Electronic supplementary material

Supplementary Information
