# Supplementary material for: Heme-dependent Inactivation of 5-Aminolevulinate Synthase from Caulobacter crescentus
Source: Sci Rep. 2018 Sep 21;8:14228. doi: 10.1038/s41598-018-32591-z (PMC6154995; doi:10.1038/s41598-018-32591-z)
Supplement: Supplementary file 1 — Supplementary Information [file 41598_2018_32591_MOESM1_ESM.pdf]

<u>Supplementary Information</u>

Heme-dependent Inactivation of 5-Aminolevulinate Synthase from *Caulobacter crescentus*

Hiroko Ikushiro, Atsushi Nagami, Tomoko Takai, Taiki Sawai,

Yuki Shimeno, Hiroshi Hori, Ikuko Miyahara, Nobuo Kamiya,

Takato Yano

Supplementary Methods

Kinetic data analysis

ALAS catalysis was shown to follow the ordered Bi-Bi mechanism in which glycine binds to the enzyme prior to succinyl-CoA and 5-aminolevulinic acid dissociates last [1] as shown in Supplementary Figure S1A.

The initial velocity equation for this mechanism was as follows[2]:

$$v = \frac{V_{max}[Gly][SucCoA]}{K_s^{Gly}K_m^{SCoA} + K_m^{SCoA}[Gly] + K_m^{Gly}[SucCoA] + [Gly][SucCoA]} \quad \cdots\cdots \quad (1)$$

where $[Gly]$ and $[SucCoA]$ are the concentrations of glycine and succinyl-CoA, respectively. K_m^{Gly} is the limiting Michaelis constant for glycine when the succinyl-CoA concentration is saturating, K_m^{SCoA} is the limiting Michaelis constant for succinyl-CoA when the glycine concentration is saturating, and K_s^{Gly} is the limiting value of the Michaelis constant for glycine when the succinyl-CoA concentration approaches zero. When $[SucCoA]$ is varied at fixed $[Gly]$, the $1/v$ coordinate for each substrate concentration in the Lineweaver–Burk double reciprocal plot is given by:

$$\frac{1}{v} = \frac{K_m^{SCoA}}{V_{max}}\left(1 + \frac{K_s^{Gly}}{[Gly]}\right)\frac{1}{[SucCoA]} + \frac{1}{V_{max}}\left(1 + \frac{K_m^{Gly}}{[Gly]}\right) \quad \cdots\cdots \quad (2)$$

In the plot of $1/v$ versus $1/[SucCoA]$, the slope and the intercept on the $1/v$ axis are respectively equal to the following:

$$Intercept = \frac{1}{V_{max}}\left(1 + \frac{K_m^{Gly}}{[Gly]}\right) \quad \cdots\cdots \quad (3)$$

$$Slope = \frac{K_m^{SCoA}}{V_{max}}\left(1 + \frac{K_s^{Gly}}{[Gly]}\right) \quad \cdots\cdots \quad (4)$$

The intercept values were then replotted versus $1/[Gly]$ to determine K_m^{Gly} and V_{max}, and the slope values were similarly replotted to determine K_m^{SCoA} and K_s^{Gly}. The non-linear regression analysis software program Igor Pro ver6.3 (WaveMetrics) was used for analysis.

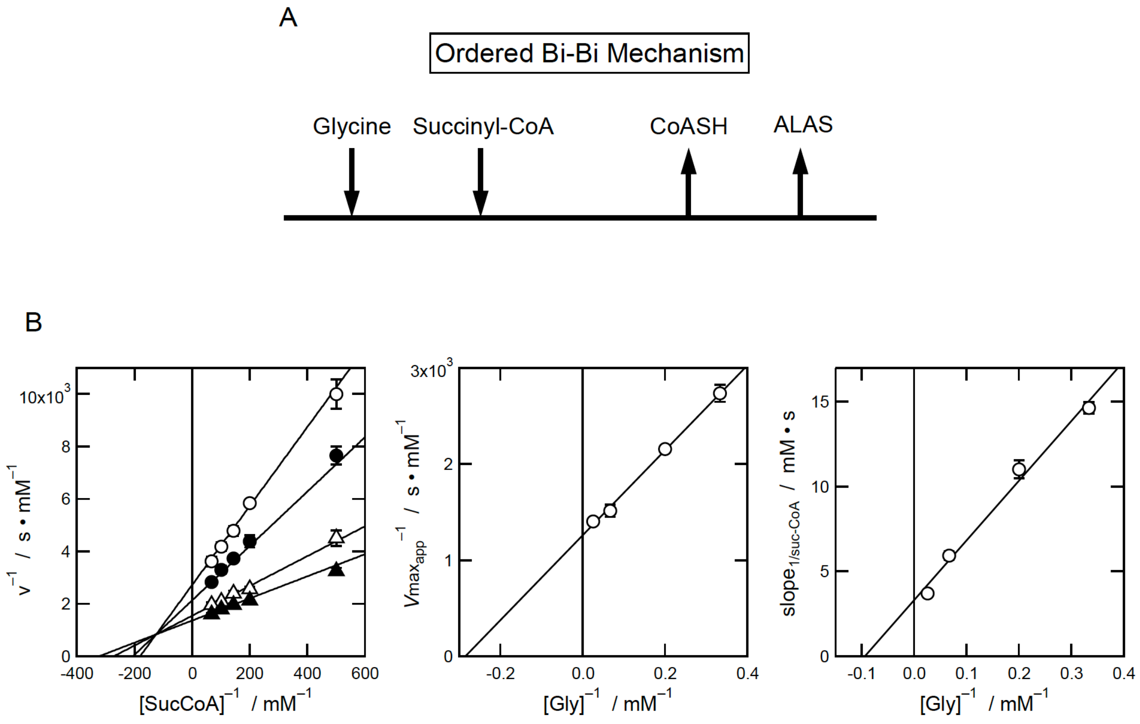

Supplementary Figure S1: Steady state kinetic analysis of wild type enzyme of cALAS. (A) Reaction scheme depicting the steady-state ordered Bi–Bi mechanism of ALAS. (B)Kinetic characterization of the PLP form of cALAS wild type enzyme. The continuous spectrophotometric enzyme-coupled assay with cALAS and α-KGD [3] was performed as described in the *Methods* section. The left panel is the primary plot of $1/v$ versus $1/[SucCoA]$ at different fixed glycine concentrations. The concentrations of glycine were 3.0 mM (open circles), 5.0 mM (closed circles), 15 mM (open triangles), and 40 mM (closed triangles). The concentrations of succinyl-CoA were 2.0, 5.0, 7.0, 10, and 15 μM. The center panel is the replot of the $1/v$-axis intercepts of the left panel versus $1/[Gly]$. The K_m for glycine was calculated from the y-axis intercept, and V_{max} was estimated from the slope. The right panel is the replot of slope of the left panel versus $1/[Gly]$. The K_s for glycine and K_m for succinyl-CoA were calculated from the y-axis intercept and the slope of the right panel, respectively. Each solid line represents the theoretical curve according to the initial velocity equation on the ordered Bi-Bi mechanism (see the *Methods* section) using the kinetic parameters summarized in Table 1.

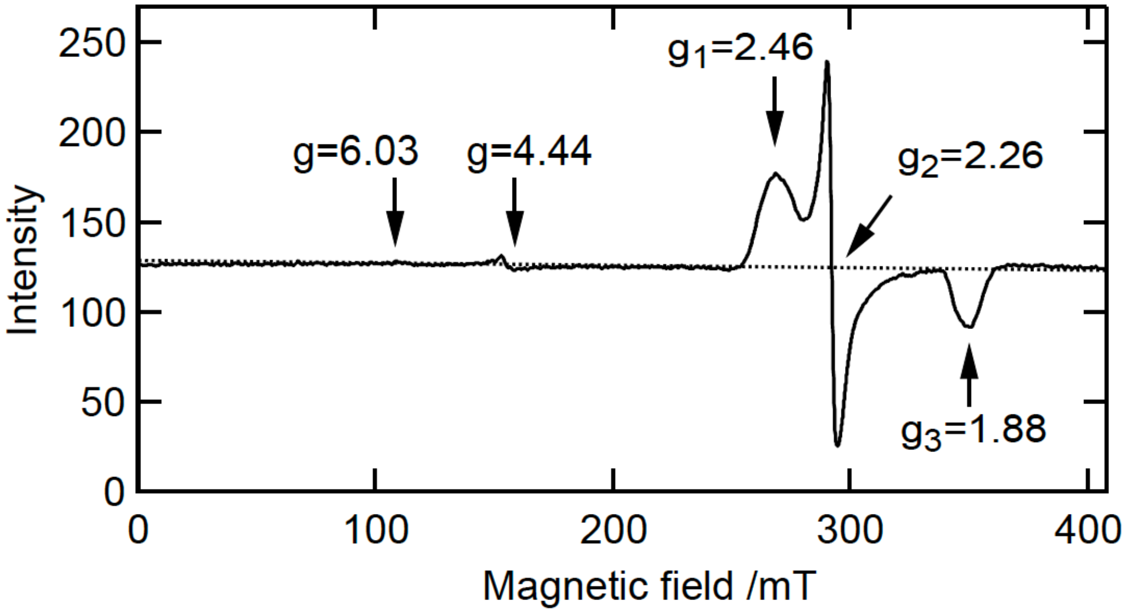

Supplementary Figure S2: EPR spectrum of the heme form of cALAS at 15 K.

Supplementary Reference

1 Fanica-Gaignier, M. & Clement-Metral, J. 5-Aminolevulinic-acid synthetase of Rhodopseudomonas spheroides Y. Kinetic mechanism and inhibition by ATP. *Eur J Biochem* **40**, 19-24 (1973).

2 Segel, I. H. Enzyme kinetics : behavior and analysis of rapid equilibrium and steady-state enzyme systems. Chap.9, 560-574 (1975).

3 Hunter, G. A. & Ferreira, G. C. A continuous spectrophotometric assay for 5-aminolevulinate synthase that utilizes substrate cycling. *Anal Biochem* **226**, 221-224 (1995).
